# Parity of Calving Influences the Likelihood of Calves Having *Cryptosporidium* spp.

**DOI:** 10.1155/2022/3306052

**Published:** 2022-03-08

**Authors:** Alīna Zolova, Dace Keidāne, Maksims Zolovs

**Affiliations:** ^1^Institute of Food and Environmental Hygiene, Faculty of Veterinary Medicine, Latvia University of Life Sciences AndTechnologies, Kr. Helmana Street 8, Jelgava LV-3004, Latvia; ^2^Department of Biosystematics, Institute of Life Sciences and Technology, Daugavpils University, Parades Street 1a, Daugavpils LV-5401, Latvia; ^3^Riga Stradins University, Statistics Unit, Balozu Street 14, Riga LV-1007, Latvia

## Abstract

The effect of colostrum on calves' health status was intensively studied, while the role of transition milk was left underestimated. The common practice is to feed calves with an adequate amount of colostrum immediately after calving and soon after feeding calves are weaned from dams. In this research, calves were not weaned from dams for at least 2 weeks receiving both colostrum and transition milk on demand. Thus, we have recreated natural feeding conditions for calves' development. We used a stratified sample method to test whether the size of the dairy cattle farms, breed, parity number, season of calving, and length of the dry period affect the likelihood of calves' infection with *Cryptosporidium* spp. considering these factors influence both colostrum and transition milk quality. The main results showed that 26.1% of calves were positive for the presence of *Cryptosporidium* spp. oocysts. The presence of clinical signs of diarrhea was recorded in 15% of the positive animals. Regression analysis showed that multiparous cows decrease the chance of calves to have *Cryptosporidium* spp. by 82%–89%, while cows calved on small farms decrease the chance of calves to have *Cryptosporidium* spp. by 80%. We suggest that primiparous cows are spending inner resources primarily on their maturation, thereby leaving the prerequisites for the infection of their offspring, while intense farming just increases the chance of unprotected calves to obtain infections.

## 1. Introduction

Colostrum is an exceptionally complex secretion that contains more than 250 various active chemical compounds [1]. For example, it contains major nutrients (fat, lactose, proteins, minerals, and vitamins) and various growth factors [2, 3] and immune factors (live maternal immune cells, antimicrobial, antiviral, and antifungal matter) [4]. Because immunoglobulin G (IgG) is the predominant antibody present in cow colostrum and because calves are born without protective antibodies and must consume colostrum immediately after birth, immunoglobulins are the best-studied components of cow colostrum [5, 6]. The ingestion of an adequate volume of high-quality colostrum is one of the most important factors influencing the health and survival of dairy calves because it provides passive transmission of immunity from cow to calf. Although, IgG is reported as a protective substance [7] against various pathogens (*Yersinia enterocolitica, Campylobacter jejuni, Escherichia coli, Klebsiella pneumoniae, Serratia marcescens, Salmonella typhimurium, Staphylococcus, Streptococcus,* and *Cryptosporidium*), Derbakova et al. [8] have not recorded a relationship between the level of IgG in bovine colostrum and the likelihood of *Cryptosporidium* spp. infection in calves. *Cryptosporidium* is a microscopic parasite that causes neonatal diarrhea in calves, resulting in a substantial economic loss to animal husbandry [9].

Because, in addition to IgG, there are many other factors in colostrum that may potentially influence infection with *Cryptosporidium* spp. [10], it seems reasonable to evaluate factors that affect the quality of colostrum and their relationship to *Cryptosporidium* spp. infection. Moreover, the quality of colostrum depends on many factors such as cow age [11], breed [12, 13], parity number [13], calendar season [14], and length of dry period [15, 16]. Soon after colostrum secretion, cows produce transition milk for 1–2 days whose properties are lower than colostrum but higher than mature milk [17, 18]. However, Kargar et al. [19] suggest that extended transition milk feeding for 3 weeks improves growth performance and reduces the susceptibility to diarrhea in calves.

In light of this, this study aimed to test the association between *Cryptosporidium* spp. infection in calves and such factors as the size of the dairy cattle farms, breed, parity number, season of calving, and length of the dry period. The research objects were calves not weaned from dams for at least 2 weeks receiving both colostrum and transition milk on demand.

## 2. Materials and Methods

### 2.1. Sample Collection and Examination

Because many dairy cattle farms separate calves from cows soon after birth, we calculated the required minimum sample size for regression analysis with multiple factors according to Green's [20] recommendation: 90 calves was the minimum sample size needed for this study. A stratified random sampling method was used to collect the data. The fecal samples were collected by veterinarians from the rectums of calves between December 2018 and December 2020. All coprological samples were exanimated on the collection day. Laboratory examinations were conducted in the Laboratory of Parasitology, Institute of Food and Environmental Hygiene, Faculty of Veterinary Medicine, Latvia University of Life Sciences and Technologies.

Totally, fecal samples were obtained from 153 calves. Fecal samples were collected from 15 ± 2 day old calves who received colostrum of ∼2.5 L within the first 4 hours of life (supervised or assisted where necessary), ∼4 L within the first 12 hours of life and then continued receiving transition milk within 2 weeks. Samples were collected in disposable polyethylene packages and stored in a transportable cooler during transport to the laboratory until examined. To detect oocysts of *Cryptosporidium* spp. in feces, the flotation method was used according to Fujino et al. [21]. Slides were stained using the modified Ziehl-Neelsen method [22]. All procedures performed in studies involving animals were in accordance with the ethical standards. The study was approved by the Animal Welfare and Ethical Council of the Faculty of Veterinary Medicine, Latvia University of Life Sciences and Technologies, and complied with current laws in Latvia.

### 2.2. Questionnaire

Before fecal samples of calves were collected, the dairy farm owner was asked to fill in the anonymous questionnaire. The questionnaire did not contain questions about personal data, as this information was not collected in any other form. There were the following questions with classified answers: (1) size of dairy cattle farm: small (≤10 cows), medium (11–50 cows), and large farm (>50 cows); (2) cow breed; (3) parity number: 1, 2, and ≥3; (4) calendar season of calving: winter (December, January, and February), spring (March, April, and May), summer (June, July, and August), and autumn (September, October, and November); (5) dry period length: ≤45, 46–64, and ≥65 days. The obtained data was used to build regression model.

### 2.3. Statistical Analysis

Generalized linear mixed modelling was conducted to determine whether explanatory variables (size of dairy cattle farm, breed, parity number, calendar season of calving, and dry period length) are related to the probability of occurring calves' infection with *Cryptosporidium* spp. where farm identification number (“FarmID”) was set as a random effects variable. Akaike's information criteria (AIC) were used to evaluate which model better fits the data. The prevalence of parasites was calculated as the percentage of hosts infected by *Cryptosporidium* spp. Statistical data analysis was conducted using Jamovi version 2.0.0 [23].

## 3. Results

**Figure 1:**
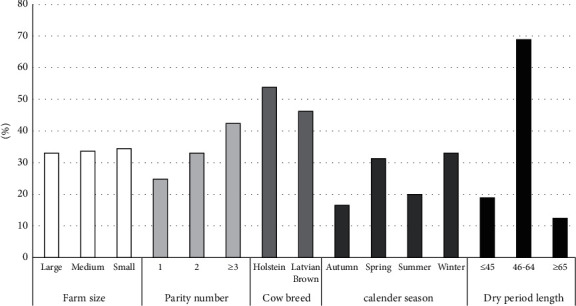
The proportion of categories of explanatory variables describing examined fecal samples of calves.

Out of all the fecal samples analyzed, 26.1% of calves were positive for the presence of *Cryptosporidium* spp. oocysts. The presence of clinical signs of diarrhea was recorded in 15% of the positive animals. The proportion of categories of explanatory variables was summarized and visualized in Figure 1. Generalized linear mixed modelling revealed a statistically significant effect of parity (*X*^2^(2) = 15.83 *p* < 0.001) and farm size (*X*^2^(2) = 8.68 *p*=0.013) on the likelihood of *Cryptosporidium* spp. infection in calf. The second cow calving significantly predicted the chance of infection of *Cryptosporidium* spp. (*B* = −1.723, *z* = −3.073, *p*=0.002, OR = 0.18). This indicates that cows having their second calving decrease calves' chances of having *Cryptosporidium* spp. by 0.18 times (or by 82%) on average, 95% CI [0.05–0.54] compared to cows having their first calving. The third cow calving significantly predicted the chance to occur infection of *Cryptosporidium* spp. (*B* = −2.181, *z* = −3.71, *p* < 0.001, OR = 0.11). This indicates that cows having their third calving decrease calves' chances of having *Cryptosporidium* spp. by 0.11 times (or by 89%) on average, 95% CI [0.03–0.36] compared to cows having their first calving. The small farm size significantly predicted the likelihood of *Cryptosporidium* spp. infection (*B* = −1.624, *z* = −2.843, *p*=0.004, OR = 0.20). This indicates that cows having their calving on small farms decrease calves' chances of having *Cryptosporidium* spp. by 0.20 times (or by 80%) on average, 95% CI [0.06–0.60] compared to large farm size. Other factors did not show significant effect on the chance to occur infection of *Cryptosporidium* spp. in calves.

## 4. Discussion

The main results showed that the parity number, as well as farm size markedly affect the chance of calves having *Cryptosporidium* spp. infection even, they immediately received colostrum and were fed by transition milk within two weeks, whereas cow breed, calendar season, and dry period length have no effect.

Our result of *Cryptosporidium* oocyst shedding (prevalence = 26.1%) is slightly higher than the reported result from neighboring Estonia (average prevalence = 23%) [24]. They also show that prevalence is markedly higher (52.03%) for calves aged between 8 and 14 days; however, nothing is known about intake of colostrum and transition milk by calves, suggesting that young animals are more susceptible to *Cryptosporidium* spp.

Although farm size cannot directly influence colostrum or transitional milk quality, there are many indirect factors that distinguish between large and small farms. For example, farm management differs between small and large farms. Usually, large farms work as a business by employing professionals to keep animals in perfect conditions to receive the maximum outcome, whereas small farms belong to families that keep animals where some specialists such as veterinarians are outsourced. Therefore, animals receive different conditions of keeping. For example, in large farms, cows have a regimented dry period length, whereas in small farms, the dry period may be set individually depending on cow health, behavior, and other factors. On small farms, calves are born mostly in the winter and spring seasons, whereas on large farms calves are born throughout the whole year. Results of this research show that in large farms calves have higher probability to have *Cryptosporidium* spp. infection compared to small farms. It may be explained by the high animal density kept in one place, since an increase in the number of hosts affects the probability for parasite transmission stages to contact new hosts. We suggest that the high density of hosts and the specificity of large farm management play a significant role in parasite transmission. For example, Mennerat et al. [25] have also discussed in detail the evolutionary implications for parasites in the frame of intense farming.

Parity numbers have been suggested to influence colostrum composition. For example, Morill et al. [13] found that with increasing parity number, the IgG concentration increased, and somatic cell count (SCC) decreased. Gulliksen et al. [26] suggest that older cows, being exposed to antigens for a longer time during their life than younger cows, produce colostrum with higher antibody levels; however, this is not always the case [27, 28]. Colostrum is the essential source of minerals (Ca, P, Mg, Na, Fe, Zn, Cu, and Mn) for newborn calves. Its concentration is significantly higher within the first hours after parturition and markedly differs between primiparous and multiparous cows [29]. The parity number also influences the mineral status of newborn calves. For example, Kume and Tanabe [29] showed that the hematocrit (Hct) and hemoglobin (Hb) of newborn calves increased as the parity number increased, and they suggested that the low Hb of primiparous cows is related to the high Fe demands of growing cows. Also, parity number is negatively associated with the cow gestation period and positively associated with the amount of milk production and calf birth weight [30].

The quality of colostrum may vary between different cow breeds [31, 32]; however, no evidence of a breed effect on infection with *Cryptosporidium* spp. Our study also showed no relationship between *Cryptosporidium* spp. infection and cow breed. Perhaps this is because there is no obvious difference in defensiveness against pathogens between many breeds of cows [33].

Seasonal variation in infectious disease transmission plays an important role, for example, high ambient temperature and high rainfall is associated with the risk of *Cryptosporidium* infection [34]. However, we did not find the effect of seasonality on the likelihood of *Cryptosporidium* spp. infection in calves. We suggest that the conditions of keeping animals on farms are the key to the lack of such a relationship. Perhaps, animals become infected in calves' pens rather than in pasture fields where ambient factor fluctuation is common and cyclic. In addition, oocyst robustness plays an important role in infection by eliminating the negative impact of the environment on the survival of the pathogen [35], which leads to year-round infection regardless of the changing seasons.

The length of the dry period influences the following properties: the amount of milk and colostrum production, IgG concentrations in colostrum, the risk of mastitis, postpartum metabolic disorders of the cow, ruminal flora development of the cow, and the energy balance of the cow [15, 16, 36–38]. There is no evidence of the effect of dry period length on the health status of calves [39], although colostrum from cows with a short dry period has a lower IgG concentration compared with colostrum from cows having a long dry period [15]. We did not find a relationship between the length of the dry period and the likelihood of *Cryptosporidium* infection. However, we do not exclude that our result was influenced by the fact that 69% of dams had ∼8-week dry period. In Latvia, farmers rarely shorten or extend the dry period of the cow.

## 5. Conclusion

In conclusion, evidence of parity relation to IgG, somatic cell count, source of minerals in colostrum, produced amount of mature milk, calves' birth weight, hematocrit and hemoglobin of newborn calves [13, 26, 29, 30], as well as the chance of calves having *Cryptosporidium* spp. infection seems to indicate that primiparous cows are spending inner resources primarily on their maturation, thereby leaving the prerequisites for the infection of their offspring, while intense farming just increases the chance of unprotected calves to obtain infection.

## Data Availability

The data used to support the findings of this study are available from the corresponding author upon request.
